# Mutant myocilin impacts sarcomere ultrastructure in mouse gastrocnemius muscle

**DOI:** 10.1371/journal.pone.0206801

**Published:** 2018-11-05

**Authors:** Jeffrey M. Lynch, Andrew J. Dolman, Chenying Guo, Katie Dolan, Chuanxi Xiang, Samir Reda, Bing Li, Ganesh Prasanna

**Affiliations:** Ophthalmology, Novartis Institutes for BioMedical Research, Cambridge, MA, United States of America; University of Iowa, UNITED STATES

## Abstract

Myocilin (*MYOC*) is the gene with mutations most common in glaucoma. In the eye, MYOC is in trabecular meshwork, ciliary body, and retina. Other tissues with high MYOC transcript levels are skeletal muscle and heart. To date, the function of wild-type MYOC remains unknown and how mutant MYOC causes high intraocular pressure and glaucoma is ambiguous. By investigating mutant MYOC in a non-ocular tissue we hoped to obtain novel insight into mutant MYOC pathology. For this study, we utilized a transgenic mouse expressing human mutant MYOC Y437H protein and we examined its skeletal (gastrocnemius) muscle phenotype. Electron micrographs showed that sarcomeres in the skeletal muscle of mutant CMV-MYOC-Y437H mice had multiple M-bands. Western blots of soluble muscle lysates from transgenics indicated a decrease in two M-band proteins, myomesin 1 (MYOM1) and muscle creatine kinase (CKM). Immunoprecipitation identified CKM as a MYOC binding partner. Our results suggest that binding of mutant MYOC to CKM is changing sarcomere ultrastructure and this may adversely impact muscle function. We speculate that a person carrying the mutant *MYOC* mutation will likely have a glaucoma phenotype and may also have undiagnosed muscle ailments or vice versa, both of which will have to be monitored and treated.

## Introduction

Trabecular meshwork induced glucocorticoid response protein (TIGR) was identified almost 20 years ago by two independent groups [1, 2] desiring to understand genes upregulated following dexamethasone treatment, since steroid-induced glaucoma constitutes a major subset of glaucoma patients. At the time, Northern blots suggested high expression of TIGR in the eye as well as expression in skeletal muscle and heart [3–5]. Given the tissue distribution and considering that the N-terminal of the protein shares approximately 25% identity with myosin, *TIGR* was later renamed myocilin (*MYOC*). To date, *MYOC* is the gene with mutations most strongly-linked to glaucoma and is reported in approximately one-third of all juvenile open angle glaucoma (JOAG) patients [6] and up to 4% of all primary open angle glaucoma (POAG) cases [7, 8]. More than 70 pathological MYOC mutations have been reported and most are found in the C-terminal of the protein (refer to http://www.myocilin.com). The C-terminal of MYOC contains an olfactomedin (OLF) domain and shares 40% identity with the nearest OLF family member. Similar to most OLF family members, myocilin is a secreted protein, but MYOC with C-terminal pathological mutations are not secreted *in vitro* [9]. Despite intense study (for review see [10]), it is unknown definitively how mutant MYOC causes glaucoma and the function of wild-type (wt) MYOC has remained elusive.

Several mouse models over-expressing wt MYOC or MYOC mutant proteins have been established to study intraocular pressure (IOP) and glaucoma disease development [11–14]. Although the eye and glaucoma have been the primary focus when studying pathological MYOC mutations, there is interest in knowing if *MYOC* mutations result in pathology in other tissues. Patients with POAG and a mutation in the *MYOC* gene have been reported to be phenotypically similar to other POAG patients without a *MYOC* mutation [15]. In 2002, Tamm stated that it was remarkable that patients with pathological *MYOC* mutations were at high-risk for glaucoma, but apparently had no other disease [10]. Could this be an area that has been overlooked? As such, studying MYOC in other tissues could provide missing insight into MYOC biology. Additionally, knowledge gained by studying myocilin in other tissues may assist physicians in early identification of patients suspected to carry a pathologic *MYOC* mutation.

Myocilin transcripts are high in muscle [3–5] and a BAC transgenic mouse with 15-fold over-expression of wt mouse MYOC protein was reported to have skeletal muscle hypertrophy with an approximate 40% increase in gastrocnemius muscle weight [16]. Thus, it is possible that MYOC is impacting cells in tissues other than those of the eye. Our present study is the first to examine the impact of over-expressing MYOC with a pathologic mutation in skeletal muscle. We utilized a transgenic mouse with CMV-driven expression of cDNA encoding for the human MYOC Y437H mutant protein [14], which in humans is a severe *MYOC* mutation associated with JOAG [7, 17]. In the skeletal (gastrocnemius) muscle of these transgenic mice, we did not observe evidence of sarcoplasmic/endoplasmic reticulum (SR/ER) stress associated with mutant MYOC nor did we observe muscle hypertrophy; however, there is a novel phenotype pertaining to the sarcomere M-line suggestive that there is compromised sarcomere integrity. We found that CMV-MYOC-Y437H transgenic mice had reduced muscle creatine kinase (CKM) a reduction of which has been reported to result in diminished exercise capacity [18]. We believe that mutant MYOC may be causing this muscle pathology through protein-protein interactions and/or due to accumulation of intracellular protein aggregates. Our findings from this transgenic animal suggest that people carrying pathological *MYOC* mutations may have a skeletal muscle phenotype. This information could aid physicians in early identification of patients carrying a pathological *MYOC* mutation and at high risk for glaucoma.

## Results

**Fig 1:**
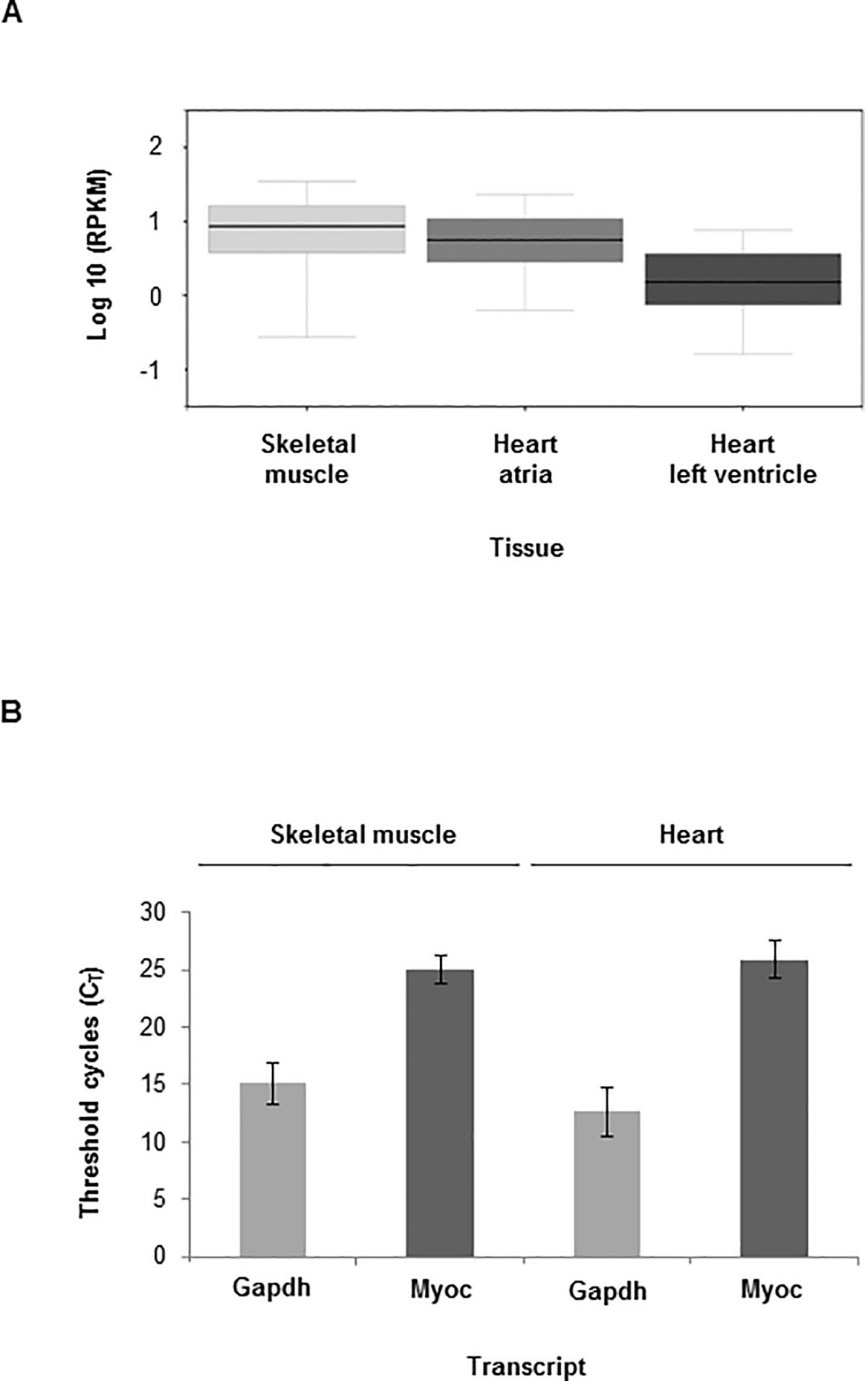
Myocilin transcript is found in skeletal muscle and heart in humans and mice. (**A**) GTEx database shows that *MYOC* gene is expressed in human skeletal muscle and heart; and (**B**) Real-time PCR (RT-PCR) confirms that Myoc transcript is in wild-type mouse skeletal muscle and heart (+/- SD). Note that the myocilin data used for Fig 1A were obtained from the GTEx Portal (https://www.gtexportal.org/home/) in May 2017.

**Fig 2:**
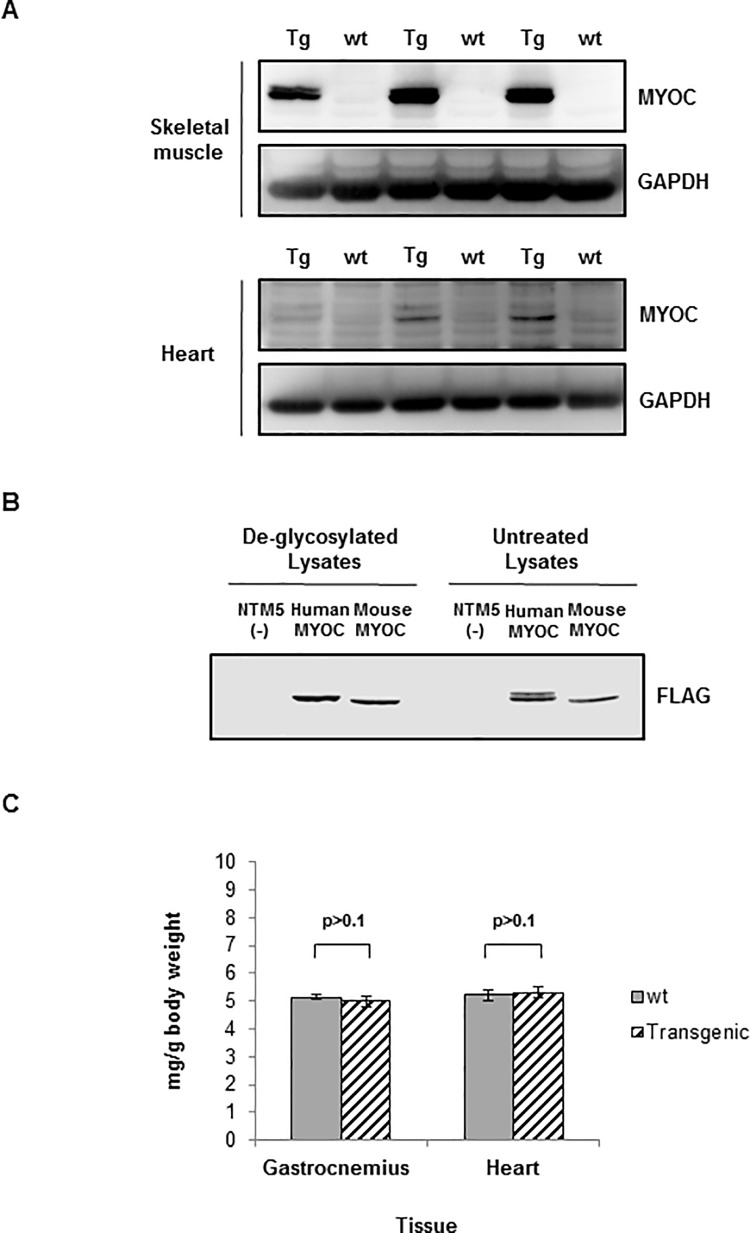
Transgenic mice had human mutant MYOC expressed in skeletal muscle and human MYOC is detected by Western blot as a doublet due to partial *N-*glycosylation. (**A**) Western blots for human MYOC protein in adult transgenic mouse gastrocnemius muscle lysates. Human MYOC protein was detected in the transgenic mouse skeletal muscle and in the heart using R&D Systems anti-MYOC antibody (1:1000). Loading was 40μg tissue lysate per well of a 10% SDS-PAGE gel. Total mouse number for this representative western blot is N = 6 different animals. (**B**) NTM5 cells were transiently-transfected with a plasmid with cDNA for human MYOC-FLAG or a plasmid with cDNA for mouse Myoc-FLAG. 20μg cell lysates were treated with PNGase to remove *N-*glycosylation followed by Western blot analysis using anti-FLAG (1:1000). (**C**) Weights of gastrocnemius muscles and hearts harvested from equal numbers of male and female wt and CMV-MYOC-Y437H transgenic mice did not differ between groups. N per group was ≥ 6. +/- SD is indicated and t-test were p>0.1. Abbreviations–wild-type, wt; transgenic, Tg.

Re-derived CMV-MYOC-Y437H mice did not have a glaucoma phenotype (S1 and S2 Figs). Based on the literature, it was anticipated that by 3 months of age the CMV-MYOC-Y437H mice would display a significant elevation in nighttime IOP (14mm Hg in wt versus 20mm Hg in transgenic) and by 12 to 14-months of age 30% of their RGCs would have been lost [14]. In the CMV-MYOC-Y437H mice we did not observe any mean IOP difference between the wt and MYOC Y437H transgenic and we did not detect a PM IOP elevation for the animals (S1 Fig). This experiment was repeated several times using different aged cohorts of animals and similar results between the groups were always obtained. In addition, we did not observe differences in axon number when comparing the wt to transgenic animals (S2 Fig). Note that there are a few dark colored axons that could be seen in images from both wt and MYOC Y437H transgenic mice; however, it is typical that even healthy optic nerve has certain axon turnover and there is no apparent difference in the incidence of these dark colored axons between the wt and transgenic. A possible explanation for our discrepancies for phenotype with that of the literature may be attributed to mouse strain [19] or epigenetics and it could be argued that, despite repeating experiments several times, had we greatly increased N values for some experiments it may have disclosed minor differences. However, our inability to detect human mutant MYOC protein in MYOC-CMV-Y437H transgenic whole eye lysates (S3A Fig) was unexpected as was our inability to detected elevated MYOC in isolated anterior eye tissues of the transgenic (S3B Fig). Furthermore, eye lysates from wt and transgenic mice did not indicate ER stress (S3B Fig). The mosaic tissue expression of the CMV-MYOC-Y437H mice had previously been noted [14], so we desired to study other tissues with mutant MYOC expression in the hope that we would gain novel *in vivo* insights into mutant MYOC pathology. We did find that the CMV-MYOC-Y437H mice expressed the human transgene in skeletal (gastrocnemius) muscle and heart (Figs 1 and 2).

Myocilin transcripts have been reported to be in adult mouse skeletal muscle and heart [4, 5] and a search of the Genotype-Tissue Expression (GTEx) database, which provides information regarding human tissue gene expression, supported this tissue expression finding (Fig 1A). Our RT-PCR results (Fig 1B) confirmed wt mouse Myoc transcript in mouse skeletal muscle (C_T_ = 25 ± 1.8) and heart (C_T_ = 26 ± 2.1). As we were unable to identify a commercial anti-MYOC antibody specific for mouse MYOC with no human MYOC cross-reactivity, we performed RT-PCR for mouse and human myocilin to estimate transcript levels in the CMV-MYOC-Y437H transgenic. The C_T_ value for human MYOC in the CMV-MYOC-Y437H transgenic skeletal muscle was found to be 19 ± 0.8 (S4 Fig). In contrast to the available anti-MYOC mouse tools, we were able to identify several commercial antibodies that work well to detect human MYOC by Western blot. As our transgenic mouse over-expressed human MYOC, we were able to identify in transgenic animals the expression of human mutant MYOC Y437H protein in both gastrocnemius muscle and heart (Fig 2A). In these tissues the human form of MYOC was found to migrate as a doublet (Fig 2A) and this doublet is due to partial *N-*glycosylation (Fig 2B). *N-*glycosylation is a post-translational modification for human MYOC [20] and is not a shared feature with mouse MYOC. The weight of the gastrocnemius muscle and the weight of the heart were found to be similar for control animals and CMV-MYOC-Y437H transgenic mice (Fig 2C), so this suggested no muscle hypertrophy.

A potential concern when studying skeletal muscle of transgenic mice is their genetic background. The SJL stain develops spontaneous myopathy due to a splice-site mutation in the Dysferlin (*Dysf*) gene which results in decreased levels of dysferlin protein and this makes the SJL stain a good model for limb girdle muscular dystrophy [21, 22]. The progressive myopathy in the SJL mice can be detected within a few weeks of age and is characterized by a progressive loss of muscle mass and muscle strength [23]. Muscle fibers in the SJL mice are replaced by fat and skeletal muscle of the SJL mice show overt histopathological abnormalities [23]. B6.SJL wt and CMV-MYOC-Y437H transgenes utilized in our study: 1) were not albino; 2) did not have less Dysf transcript (S5 Fig) or less DYSF protein (S6 Fig) in comparison to C57BL/6J mice (abbreviated herein as C57); and 3) did not have body weight or gastrocnemius muscle weight profoundly different from each other (Fig 2C) nor greatly different from the C57BL/6J mice utilized in this study (S7 Fig). Additionally, our EM images herein did not show replacement of muscle fibers with fat. Hence, our results indicate that the MYOC Y437H transgene did not adversely impact *Dysf* gene expression and having the CMV-MYOC-Y437H transgenic mice established on the B6.SJL background circumvents some concerns associated with a pure SJL background.

**Fig 3:**
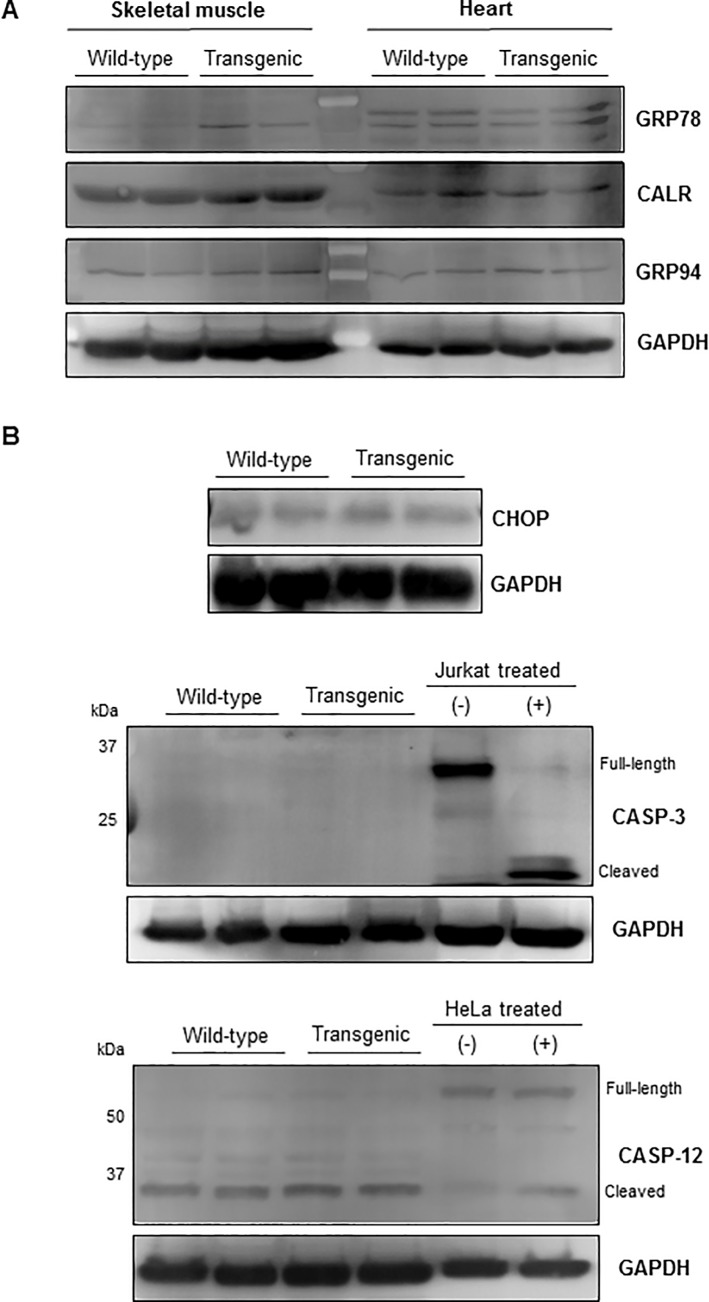
Western blots for ER protein expression in wt and CMV-MYOC-Y437H transgenic muscle lysates (40μg per lane). **(A)** Over-expression of CMV-MYOC-Y437H mutant protein in the transgenic animal has little impact on expression of ER proteins in skeletal muscle or heart. **(B)** Western blots examining CMV-Y437H mouse skeletal muscle lysates for pro-apoptotic proteins associated with ER stress showed no differences between the wt and transgenic mice. Control cell extracts for CASP-3 (10μL per well) were from Cell Signaling Technology (9663). Controls for CASP-12 were cell lysates (40μg lysates/well) obtained from HeLa cells +/- 1μM staurosporine (Abcam, ab146588) treatment for 18 hours. Note that all representative Western blots have wells loaded with lysates obtained from different animals (N = 2 wt and 2 transgenic per blot).

**Fig 4:**
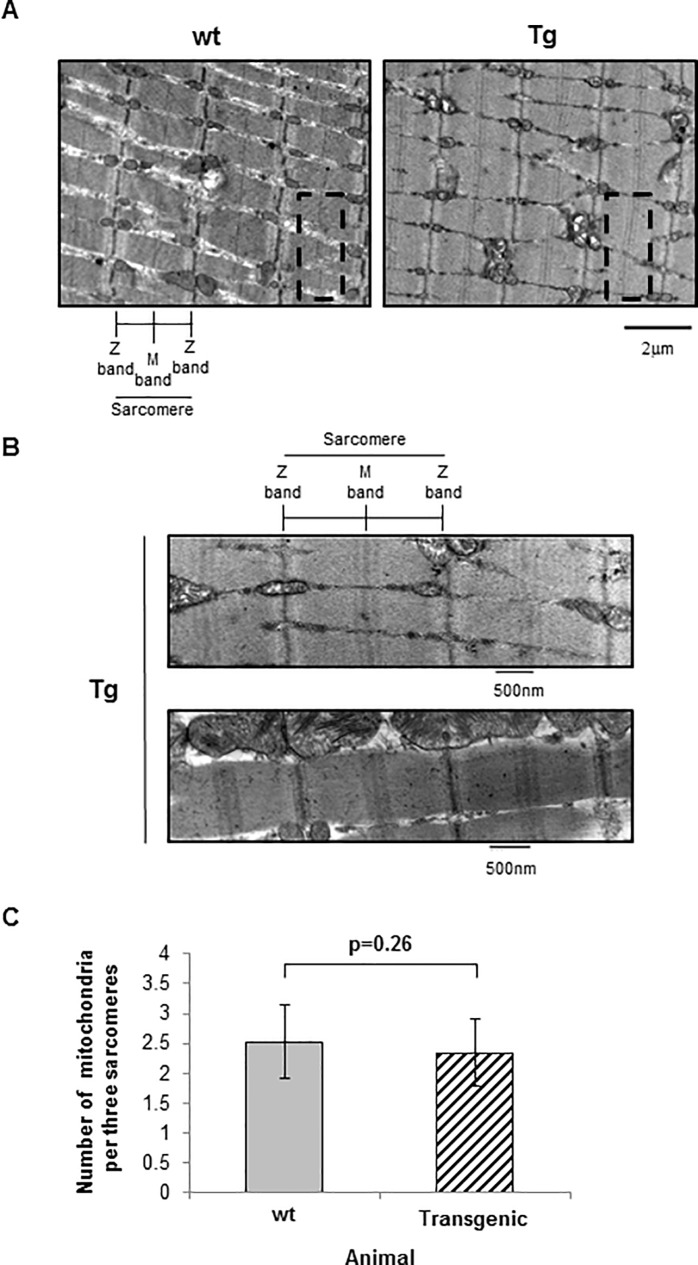
Electron micrographs of gastrocnemius muscle from 4 to 6 month old female wt and CMV-MYOC-Y437H transgenic mouse skeletal muscle. **(A)** No ER/SR expansion was observed in transgenic mouse skeletal muscle. Transgenic mouse skeletal muscle was observed to have multiple M-line bands (highlighted by dashed line box) within the H zone of the sarcomeres. Direct magnification was 4800X. **(B)** Additional electron micrographs from CMV-MYOC-Y437H transgenics have been enlarged to show the diffuse M-line in sarcomeres. Direct magnification was 18500X. **(C)** The number of mitochondria per three sarcomere was quantified from four micrographs and there was no significant difference between wt and CMV-MYOC-Y437H transgenics. +/- SD; t-test p>0.1.

Expression of mutant MYOC has been reported to produce a robust ER stress response *in vitro* [24, 25] and *in vivo* [14]. In muscle, an ER response will result in upregulation of numerous ER resident proteins [26]. Our Western blot analysis for ER proteins in the gastrocnemius muscle and heart lysates indicated a small increase in expression of GRP78 (BiP) in the transgenic, but no change was observed for CALR or GRP94 proteins (Fig 3A). This lack of uniform elevation of ER resident proteins in the transgenic does not support the concept that mutant MYOC is causing pronounced *in vivo* ER stress. Additional Western blots of the tissue lysates for pro-apoptotic proteins associated with ER stress (Fig 3B) showed no differences between the control and transgenic. As skeletal muscle is not considered a secretory tissue, we desired to know if MYOC Y437H mutant protein would show signs of severe ER stress in the form of ER expansion. To examine the ER directly, we performed Electron Microscopy of mouse gastrocnemius muscle samples. Electron micrographs of the mouse skeletal muscle showed no indication of ER expansion (Fig 4A). Therefore, we conclude that the skeletal muscle of the mutant MYOC Y437H transgenic mice showed no evidence indicative of ER stress.

Further examination of CMV-MYOC-Y437H transgenic mouse skeletal muscle EM images (Fig 4A and 4B) revealed an increase in the number of visible M-bands within the sarcomeres when compared to wt mouse muscle. This observation suggested that mutant MYOC protein expression in skeletal muscle may impact muscle ultrastructure. Mitochondria morphology is known to be highly diverse in skeletal muscle fibers [27, 28]. As the number of mitochondria and sarcomere size varied slightly among our EM images, we performed quantitative analysis. Mitochondrial number was found to be similar for both wt and CMV-MYOC-Y437H transgenics (Fig 4C).

To determine if our findings for mutant MYOC in skeletal muscle were consistent with other MYOC transgenics, we examined two human MYOC BAC transgenic mice lines, one expressing wt MYOC and the other expressing mutant Q368X MYOC (S4 Fig). Wt MYOC BAC and mutant Q368X MYOC BAC mice had MYOC transcripts observed to be in heart and gastrocnemius muscle (S4 Fig). Similar to the CMV-MYOC-Y437H transgenic, these BAC animals exhibited gastrocnemius muscle weights and heart weights comparable to control mice (S7 Fig). Electron micrographs of the gastrocnemius muscle indicated that the wt control and the wt MYOC BAC mice showed a very distinct and compact M-band. In comparison, the Q368X mutant MYOC BAC gastrocnemius muscle M-band was less defined than the controls (S8 Fig), appearing disperse and more similar to that observed for the CMV-Y437H-MYOC transgenic. H&E staining of C57 control, wt MYOC BAC transgenic, and Q368X mutant MYOC BAC transgenic gastrocnemius muscles did not indicate hypertrophy in the BAC mice (S9A and S9B Fig).

**Fig 5:**
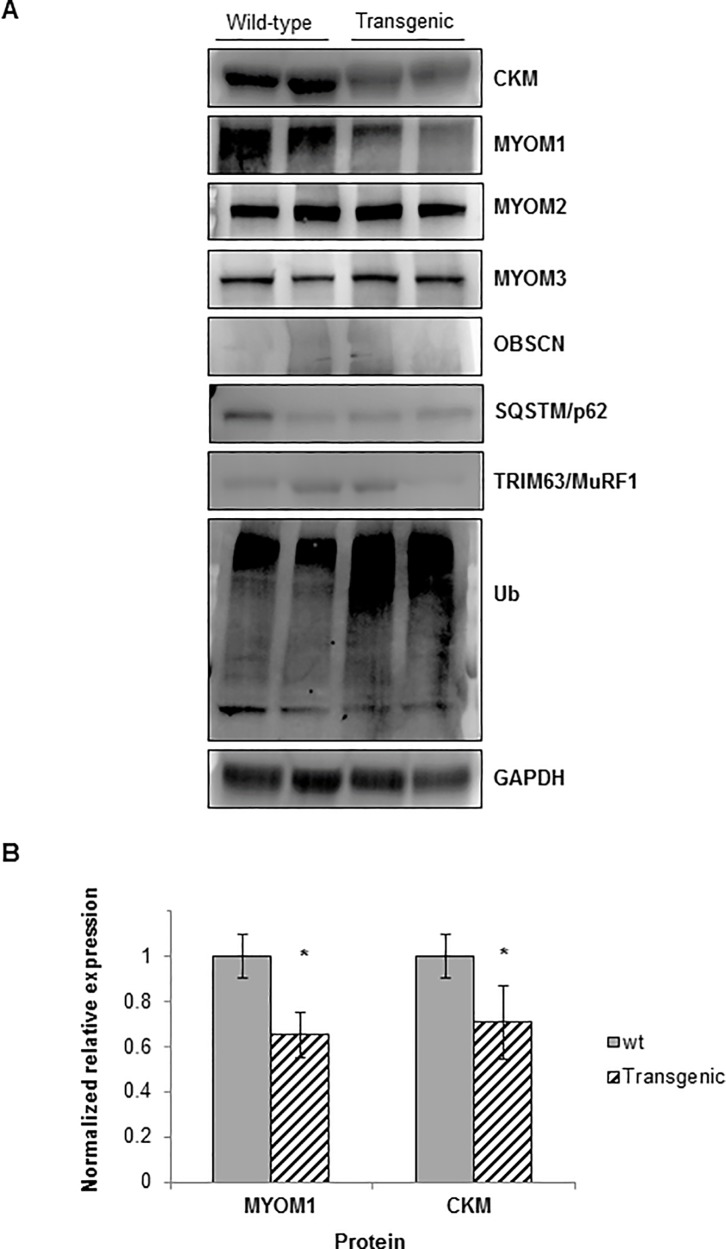
Western blots for expression of M-band proteins in wt and CMV-MYOC-Y437H mice. **(A)** Skeletal muscle lysates (40μg per lane) from MYOC Y437H mutant transgenic mice indicated lower expression of both MYOM1 and CKM in comparison to wild-type animals. Note that representative Western blot wells are loaded with tissue lysates from different animals (N = 2 wt and 2 transgenic per blot). **(B)** Densitometry was completed to measure amount of protein in MYOM1 and CKM Western blot sample lysates. Error bars are +/- SEM and * represents t-test p<0.05. Abbreviations–Ubiquitin, Ub.

**Fig 6:**
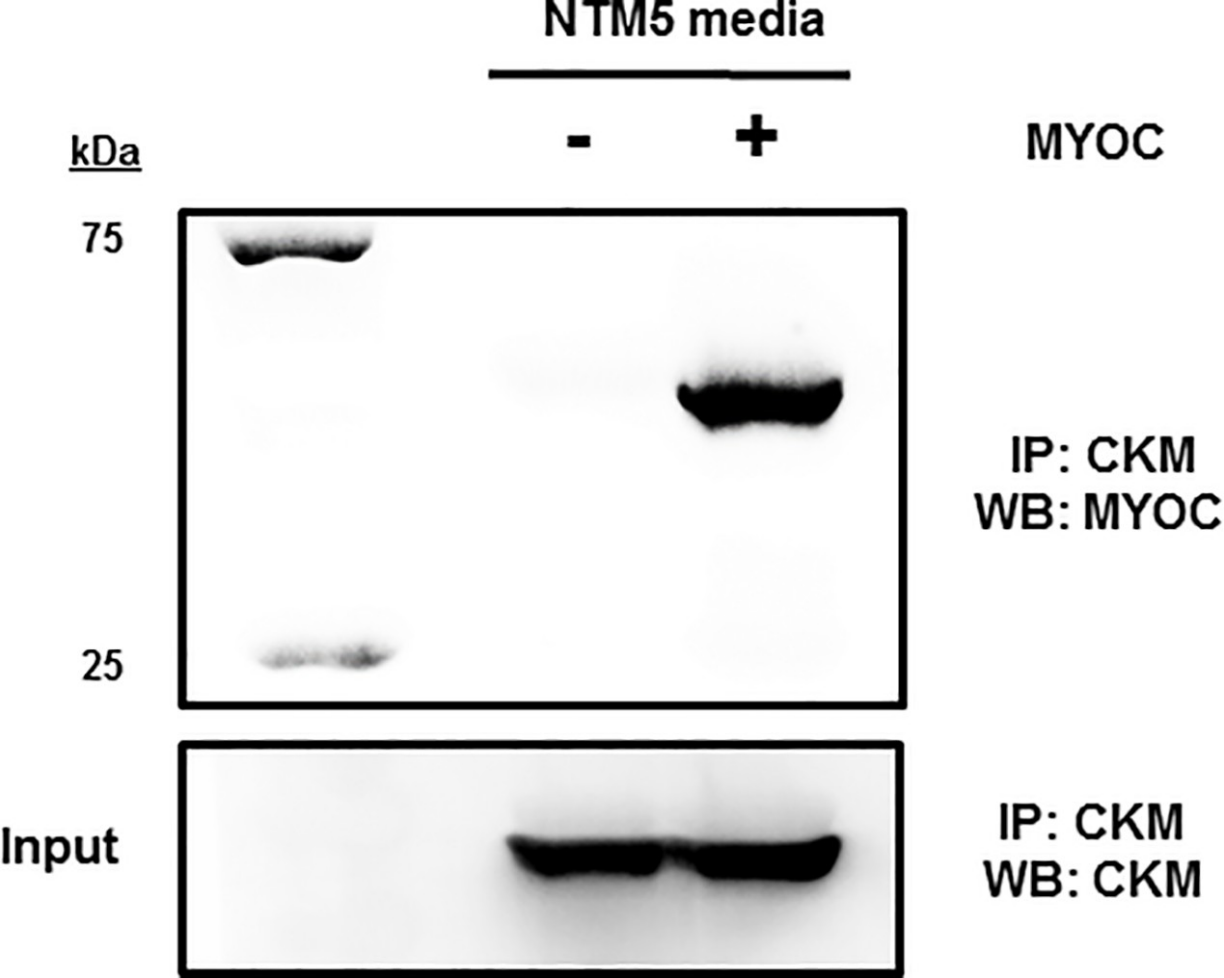
Western blot following immunoprecipitation (IP) indicates that CKM binds MYOC. The Western blot was stripped and treated with anti-CKM to show CKM input.

M-band composition consists of myomesin (Myom) family members bridged by accessory proteins such as muscle creatine kinase [29, 30]. Western blot analysis of mouse skeletal muscle for proteins typically found within the M-band showed no differences in MYOM2 or MYOM3; however, lower levels of MYOM1 protein and CKM protein were observed for the mutant CMV-MYOC-Y437H transgenic mice (Fig 5A and 5B). A yeast-two-hybrid screen using wt MYOC and a skeletal muscle library had previously been completed and reported CKM as binding MYOC [31]. We confirmed the MYOC-CKM interaction by an immunoprecipitation (IP) experiment using CKM-FLAG-tagged protein as bait. Our IP results do support a physical interaction between MYOC and CKM protein (Fig 6).

**Fig 7:**
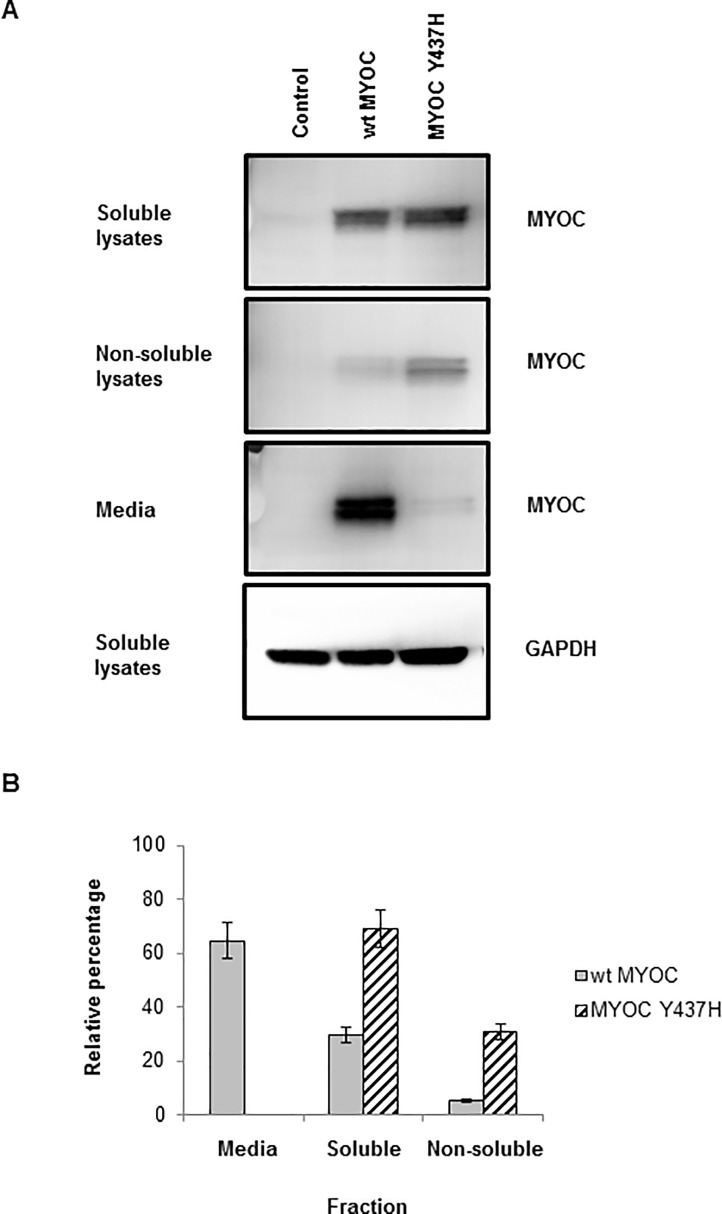
NTM5 cells transiently-transfected for 48 hours with plasmids which had MYOC cDNAs were analyzed by Western blot. (**A**) Wild-type (wt) MYOC is predominantly secreted while mutant CMV-MYOC-Y437H is not secreted and has a larger non-soluble fraction when compared to wt MYOC. Anti-MYOC goat antibody from R&D Systems was utilized. (**B**) Percentage of MYOC in each cell fraction was estimated based on Western blot band intensity. Error bars are +/-SD.

In trabecular meshwork cells, mutant MYOC is misfolded [32] and MYOC mutant protein is reported not to be secreted [9]. In our NTM5 cells transfected with MYOC cDNAs, we did observe secretion of wt MYOC as well as non-secretion of the mutant protein (Fig 7). Normally, misfolded proteins in the ER are retro-translocated to the cytoplasm and efficiently cleared by a process known as ER-associated degradation (ERAD) which utilizes the proteasome. Ubiquitin (Ub) Western blot smearing observed for the CMV-MYOC-Y37H transgenics (Fig 5A) indicates more high-molecular weight (HMW) ubiquitinated proteins relative to the wt animal which is suggestive of more misfolded protein in the transgenics. From our experiment with NTM5 cells, we did find that some of the MYOC Y437H mutant protein was detectable in the non-soluble cell fraction (Fig 7). Thus, pathology associated with mutant MYOC likely occurs due to non-secretion and aggregation of the mutant MYOC protein with itself and in complexes with other proteins.

## Discussion

There is a strong genetic link between mutant *MYOC* and glaucoma. Despite almost 20 years of intensive effort, the function of wt MYOC protein is unknown as is definitively how mutant MYOC protein contributes to disease. In the eye, MYOC protein is highly expressed in the trabecular meshwork, ciliary body, and retina and Northern blots indicate myocilin transcripts in skeletal muscle and heart [1, 4, 5]. To date, there has only been one publication regarding MYOC in skeletal muscle and that work was limited to wt mouse *Myoc* [16], so the impact of mutant myocilin on skeletal muscle had not been investigated.

The CMV-MYOC-Y437H mice [14] were acquired through a licensing agreement and the line was re-derived in accordance with CRO requirements. The data we collected from different cohorts of these MYOC transgenic mice of different ages were compared to age-matched wt animals. A limitation to our work is that we did not include a control of transgenic mice expressing CMV-wt-MYOC to compare with the mutant CMV-MYOC-Y437H mice. However, we did compare the mutant MYOC mice against wt control mice based on the established precedent in the literature [11, 12, 14] that has shown that looking at the mutant MYOC alone is sufficient to understand its biology. We found that the CMV-MYOC-Y437H mice did not have high IOP nor did the aged transgenics have an abnormal axon number. Western blots of ocular tissue lysates showed no detectable expression of the human mutant MYOC protein and this is the probable reason why these transgenics did not exhibit features of glaucoma. When we examined non-ocular tissues we found that the MYOC-Y437H transgene was expressed in gastrocnemius muscle. To gain insights into mutant MYOC biology/pathology, we further studied this muscle group. Ending of the licensing agreement meant the re-derived mice had to be terminated, so we have not conducted related experiments that may have been informative (e.g., histological studies of additional muscle groups for fiber cross-sectional area).

The literature reports a C57BL/6J mouse line with over-expression of mouse BAC wt *Myoc* [33]. This mouse line was intercrossed to produce mice homozygous for the transgene and these Tg/Tg mice exhibited a robust 15-fold wt MYOC protein over-expression [33]. In this 2004 publication, the authors did not report any overt phenotype or weight differences for these wt *Myoc* BAC transgenic mice in comparison to the wt controls. Later, this transgenic mouse with over-expression of wt mouse BAC *Myoc* was reported to have skeletal muscle hypertrophy (>40%) and a large (40%) increase in gastrocnemius muscle weight [16]. To put this muscle enlargement in perspective, myostatin-null mice have been reported to have a 47% increase in gastrocnemius muscle weight relative to wt animals [34]. In none of our MYOC transgenic mice did we observe this reported weight difference in gastrocnemius muscle. A reason for this discrepancy may due to the wt mouse *Myoc* BAC transgenic being homozygous for the transgene [16] which meant those mice had a more robust over-expression of MYOC protein in comparison to any of our study animals.

MYOC is a secreted protein which is processed in the ER and is *N-*glycosylated. Pathologic MYOC mutant proteins are not secreted [9] and are retained within the cell. It has been proposed that mutant MYOC protein *in vivo* induces severe ER stress to cause pathology [14]. We found that mutant MYOC Y437H over-expressed in skeletal muscle of transgenic mice has no major impact on expression of ER proteins. We observed a slight increase in GRP78 (BiP) expression, but we did not see a collective upregulation of numerous ER-resident proteins which would be expected for an ER stress response. Our Western blot data is predominantly qualitative, so one could speculate that there is a chance that minor differences in protein amounts could be resolved by methods more sensitive than Western blot and, if shown, these minor protein differences could potentially have biological importance. As skeletal muscle is not considered a secretory tissue, it is expected to be very sensitive to retention of a secretory protein resulting in SR/ER expansion. Our electron micrographs of CMV-MYOC-Y437H transgenic skeletal muscle did not show evidence of expanded SR/ER. Rather, we observed a novel M-band phenotype in these animals with the MYOC Y437H mutant mice appearing to have multiple M-bands within their sarcomeres. Multiple M-bands can be seen in an EM image in the literature [35], but has not been suggested as a phenotype until now. Interesting, in the literature, the appearance of multiple M-bands is only seen in the diseased tissue (i.e. dog myocardium).

The M-band is a distinct and dense protein structure at the center of the sarcomere of skeletal and cardiac muscle. The M-band is comprised of bridged M-lines and the ultrastructure correlates with contraction speed and muscle type suggesting that the M-band components have a significant physiological role [36]. Electron microscopy has shown that muscle fibers at high degrees of stretch can have the M-band become faint or undetectable within the sarcomere while in muscle fibers with extremely shortened sarcomeres the M-lines can have distinct subdivisions appearing as multiple M-bands [37]. M-bridges connect the M-lines to the myosin rods and serve to maintain thick filament alignment across the sarcomere during muscle contraction. In addition to myosin rods, numerous other proteins can be found within the M-band region and these proteins can contribute to numerous cellular activities including cytoskeletal remodeling, signal transduction, mechanosensing, metabolism, and proteasome degradation (for review see [38]). To date, the most well characterized M-band proteins are obscurin, myomesins, and muscle creatine kinase (CKM).

Obscurin contributes to the assembly and stabilization of the M-region by linking myomesin to the SR [38]. Obscurin (Unc-89 in Drosophila) is a M-line protein and in a knockdown Drosophila model the result is a missing sarcomere M-band and an inability of adults to fly [39]. Similarly, obscurin-deficient mice were found to lack the M-band and had compromised exercise endurance with the diaphragm being the skeletal muscle most severely affected [40]. These results suggest that M-band proteins are necessary to maintain structural integrity of the skeletal muscle fibers. We were not able to adequately detect obscurin by Western blot (Fig 5), so we focused on the other M-band proteins.

The major constituent of the M-band is myomesin. The function of the M-line myomesin proteins is to stabilize the sarcomere. Myomesins are structural proteins of the M-line that form an elastic structure [41] that interacts with titin and myosin [29, 42, 43]. *Myosin* genes are regulated by myocyte enhancer factor 2c (MEF2C) which is a muscle-specific transcription factor [44]. In skeletal muscle of *Mef2c*-null mice, the myomesin genes were found to be downregulated and EM images from these mice suggested that skeletal muscle myofibers deteriorated due to a loss of M-line integrity [44]. An increase in M-region cross-sectional area is postulated to enhance stiffness of the M-bridges [36]. In the M-line, MYOC could be impacting the stiffness of the sarcomere and/or the packing of myomesin and/or how the M-line is associated with the cell cytoskeleton. CKM localizes at the M-band due to interactions with myomesin [29, 45] and it is hypothesized that CKM may serve both structural as well as enzymatic functions [36]. *Ckm*-null mice exhibit decreased voluntary running ability and a decrease in force production due to an inadequate supply of local ATP [18, 46, 47, 48]. Electron micrographs of longitudinal sections through myofibers of *Ckm*-null mouse gastrocnemius muscle showed less intense M-band staining [18]; thus, CKM protein is essential for M-band cross-bridge formation.

**Fig 8:**
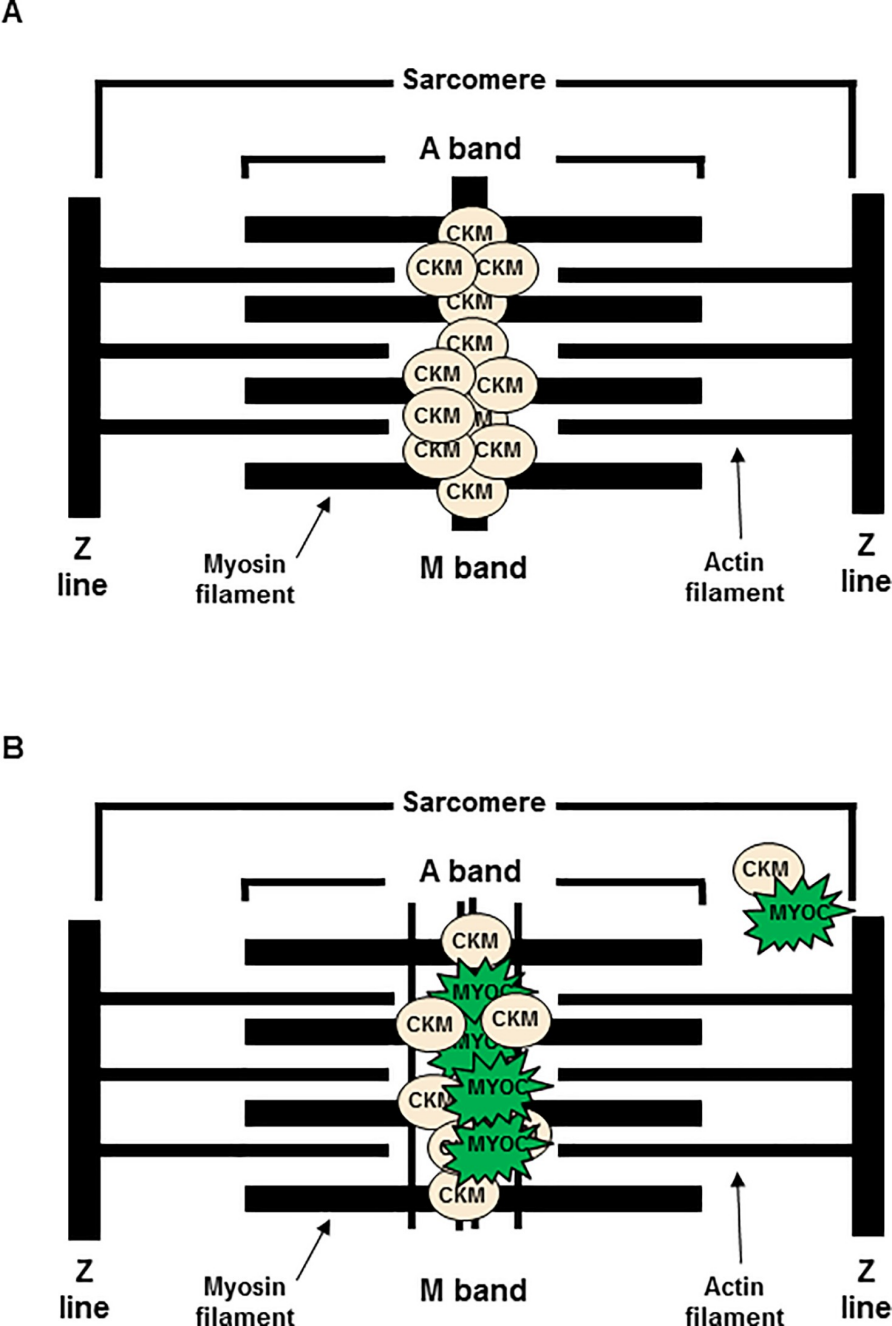
Model of mutant MYOC pathology in mouse skeletal muscle sarcomere. (**A**) Wt sarcomere has CKM binding myomesins (MYOMs) to form a dense M-line. (**B**) Mutant MYOC can bind CKM causing the M-line to become disperse. It is possible that there may be accumulation of CKM bound to MYOC at a location beyond the M-band region.

We examined by Western blot the M-band proteins in the CMV-MYOC-Y437H mutant transgenic animals and found the transgenics to have less MYOM1 and less CKM protein in comparison to wt littermates (Fig 5A and 5B). A decrease in CKM activity has been suggested to be a contributor to gradual loss of muscle function associated with aging [49]. This data suggests that the M-band phenotype may arise due to diffusion of the original and compact M-band (Fig 8). It is possible that mutant MYOC Y437H is contributing to this phenotype by: 1) binding CKM thereby disrupting the M-band normal bridging; and/or 2) through mutant MYOC protein aggregation with proteins found in skeletal muscle needed for normal sarcomere structure or maintenance. As this M-band phenotype only appears in literature for diseased tissue [35], it is highly probable that the change in M-band architecture is a physiological adaptation and may have an adverse impact on normal/optimal skeletal muscle function. These differences in muscle ultrastructure could have functional consequences and impact the animals’ responses and/or behavior. *In vivo*, 1) the MYOC Y437H transgenic animals were observed to be more irritable/aggressive in comparison to wt littermates; and 2) most transgenic mice did not recover from isoflurane exposure (Bing Li, observations). Tissue distribution of M-line proteins is limited to muscle, so it is likely that the CKM and MYOC association is unique to muscle sacromeres and would not be a factor in pathology of trabecular meshwork cells.

Skeletal muscle deteriorates in both size and strength with age [50, 51]. In rats with reduced physical activity, it is reported that Myoc transcript increases in skeletal muscle with age [52]. As reduced exercise capacity can occur due to loss of CKM [18], it is likely that the CMV-MYOC-Y437H mutant mice also have a physiological phenotype. Future investigations to determine the potential impact of this ultrastructure change on muscle function could provide valuable insight into people who carry a pathological *MYOC* mutation. Do these people have diaphragm issues or complications associated with anesthetics? Do these people exhibit modified exercise capacity or experience excessive muscle pain/stiffness? If people with a pathologic *MYOC* mutation have a skeletal muscle phenotype, this information may aid physicians in early identification of those at high risk for glaucoma.

In the cell, mutant MYOC can interact with other proteins and these interactions could impact cell integrity as well as compromise cell structure and/or function. In humans, data from the GTEx database suggests that MYOC is expressed in non-ocular tissues. People carrying a pathologic *MYOC* mutation are at extremely high-risk to develop glaucoma [53], and information that contributes to early identification of these individuals is essential for immediate intervention to help limit the impact of this devastating and blinding disease. The results we have presented in this report suggest that physicians should not only consider a patient’s family glaucoma history, but also consider the patients’ muscle ailments as a potential indication of a pathologic *MYOC* mutation and recognize the necessity for genotyping and consultation with Ophthalmologists.

## Materials and methods

### CMV-MYOC-Y437H mice

Mutant CMV-MYOC-Y437H mice have previously been described [14] and these animals were obtained through a licensing agreement. Preliminary experiments (data not shown) were completed using these animals. Maintenance of the line was transferred to a contract research organization (CRO) that rederived CMV-MYOC-Y437H mice in the B6.SJL background. All experiments were performed with F3 and later generations of intercrossed mice. Mice were genotyped as described and wt littermates served as controls [14] for the CMV-MYOC-Y437H transgenic mice. All animal experiments were performed in accordance the Association for Research in Vision and Ophthalmology (ARVO) policy on the Use of Animals in Vision Research and all protocols were reviewed and approved by the Institutional Animal Care and Use Committee (IACUC) at Novartis Institutes for Biomedical Research. All animals were housed in rooms in which the temperature, humidity, and lighting (12h:12h light-dark cycle) were controlled and water and food were available *ad libitum*. Harvested tissue samples were stored at– 80°C until utilized.

### MYOC wt BAC & MYOC Q368X BAC mice

Bacterial artificial chromosome (BAC) containing the human *MYOC* gene (RP11-1152G22) was utilized to create the BAC transgenic mouse lines. This BAC contains the complete *MYOC* coding sequence, with 68 kb 5’ and 51 kb 3’ flanking sequences. A modification of the BAC to introduce a C>T mutation at position 1102 of the coding sequence was done by GeneBridges (Heidelberg, Germany). Purified BAC constructs were injected into the pronuclei of C57BL/6J mice. The offspring were screened by PCR to identify transgenic founders and Western blot analysis confirmed expression of the human MYOC protein in these mouse lines. One founder for each of the wt MYOC BAC and mutant Q368X MYOC BAC were used to establish transgenic lines for study. No breeding complications or viability issues were noted for any of the BAC mice. All experiments were performed with F3 and later generations of intercrossed mice. All BAC mice were studied as hemizygotes with their wt littermates serving as controls.

### Measurement of conscious IOP

Animals underwent training of conscious IOP measurement for more than three weeks. IOP was measured with a TonoLab rebound tonometer (Colonial Medical Supply, Franconia, NH) twice a week at same time of day (8:00am to 11:00am and 3:30pm to 6:30pm). Ten measurements per eye/time were deemed reliable by internal software, to generate and display an average IOP reading. An average of these readings was then calculated and was reported as calculated mean IOP. Animal cohorts for IOP typically contain >10 animals per group (never less than N = 6/group) and multiple examiners perform the IOP measurements. Note that normal variability expected for multiple examiners using tonometers is approximately 1 mm Hg [54].

*Optic nerve semi-thin cross-section and paraphenylenediamine (PPD) staining—*Mouse optic nerve samples were collected and fixed with half-strength Karnovsky’s fixative (2% formaldehyde + 2.5% glutaraldehyde, in 0.1M sodium cacodylate buffer, pH 7.4 (Electron Microscopy Sciences) for a minimum of 48 hours. After fixation, samples were rinsed with 0.1M sodium cacodylate buffer, post-fixed with 2% osmium tetroxide in 0.1M sodium cacodylate buffer, then dehydrated with graded ethyl alcohol solutions, transitioned with propylene oxide and resin infiltrated in tEPON-812 epoxy resin (Tousimis) utilizing an automated EMS Lynx 2 EM tissue processor (Electron Microscopy Sciences). Processed tissues were oriented in tEPON-812 epoxy resin and polymerized in silicone molds using an oven set for 60°C for 48 hours. Cross-sections were cut at 1-micron with a Histo-diamond knife (Diatome) on a Leica UC-7 ultramicrotome (Leica Microsystems) and collected on slides then dried on a slide warmer. Cross-sections were collected at ~1mm distance posterior to the optic nerve head. The slides were stained with 2% aqueous PPD (MP Biomedicals LLC) solution for 45 minutes at room temperature, rinsed in tap and deionized water solutions, air-dried, then mounting medium and a glass coverslip was applied over the sections for light microscopic analysis of myelinated axon analysis.

### Axon quantification

Optic nerve cross sections (1μm thickness) were cut at the location of 1mm from the optic nerve head and processed for PPD stain of myelin as described in the method. Nine (110μm X 82μm) square areas were sampled per nerve for axon quantification as depicted in the schematic (S2 Fig). The number of axons was counted using ImageJ [ImageJ → Open image → Convert image type to 8-bit → Adjust image threshold (choose “Otsu” “B&W” “Dark background”) → Analyze (choose “Analyze particles” and set show “outlines”, “Display results”, “Exclude on Edges”, “In situ show”]. Mean axon density was obtained as the average of the axon count of the nine square areas. Total area of the optic nerve cross section was measured by ImageJ. Total number of axons was calculated as mean axon density multiplied by the total area. Although this axon quantification method is published [55] and is similar to other accepted quantification techniques [56], a limitation of this automated counting method is that we did not formally test it for validation against manual counts. As such, there is potential that a technical glitch could prevent us from detecting a loss of axons indicative of a glaucoma phenotype, but given the past success of this tool and the qualitative appearance of the nerves, we consider this unlikely.

### RNA isolation & RT-PCR

Skeletal muscle (gastrocnemius muscle) harvested from 4-month-old wild-type (wt) mice was minced with scissors and homogenized (Omni Tissue Master 125 Homogenizer, Omni International) and RNA isolated using a RNA isolation kit (Qiagen, 74704) according to manufacturer instructions. RNA concentration was determined using a ThermoScientific NanoDrop 2000. For Real-time PCR (RT-PCR), an Applied Biosystems ViiA7 real time PCR system (Life Technologies) was utilized. Additional materials were TaqMan RNA to C_T_ 1-step kit (Applied Biosystems, 4392938) and TaqMan Gene Expression Arrays (Applied Biosystems) for human MYOC (Hs00165345), mouse Myoc (Mm00447900_m1), mouse Gapdh (MM99999915_g1) and mouse Dysf (Mm00458050_m1). Samples were analyzed in triplicate.

Reverse-transcriptase PCR for mouse Dysf was completed using SuperScript IV One-Step RT-PCR System (Invitrogen, 12594025) according to manufacturer instructions and a Bio-Rad C1000 Touch Thermal Cycler. PCR primers to distinguish wt and mutant mouse Dysf have previously been described [21] and were synthesized by ThermoFisher Scientific. A 10μL aliquot of the PCR product had 6X Gel Loading Dye added (Cell Signaling Technology, B7021S) and each sample was loaded into wells of a 0.8% Agarose / 1X TAE gel. DNA standard was 1 Kb Plus DNA Ladder (Invitrogen, 10787–018). Agarose gels were imaged using a Bio-Rad GelDoc XR+ instrument. The wt Dysf PCR product is 500bp while the Dysf of the pure background SJL mouse is 329bp [21].

### Tissue culture and transfection conditions

A normal trabecular meshwork human cell line (NTM5) has previously been described [57] and was utilized in this study. NTM5 cells were grown in 10cm culture dishes in a 37°C incubator with 90% relative humidity and 5% CO_2_. Cell media was DMEM (Gibco, 11995–065) supplemented with 10% FBS (Gibco, 10082147) and 1% P/S (Gibco, 15140–122). NTM5 cells at 70 to 80% confluence were transiently-transfected with FLAG-tagged plasmids (8μg total cDNA per 10cm plate), human MYOC (Origene, RC206556; Accession number NM_000261), or mouse Myoc (Origene, MR224777; Accession NM_010865) using FuGENE6 transfection reagent (Promega, E2691). All plasmids had been purified using a Qiagen plasmid maxi kit (Qiagen, 12163) and for transfections FuGENE6 was used at a 5:1 ratio with the cDNA. 48-hours post-transfection, cells were washed with 1X PBS (Gibco, 20012–027) and lysed on ice using a RIPA buffer (50mM Tris pH 7.5, 150mM NaCl, 1mM EDTA pH 8.0, 1mM EGTA pH 8.0, 0.1% SDS, 1% Triton X-100, 0.5% NaDOC, 1mM DTT) with Complete protease inhibitors (Roche, 11873580001). Cell debris was removed by centrifugation at 4°C (Eppendorf 5810R). Protein assay (Bio-Rad DC kit, 500–0113, 500–0114, 500–0115) was completed in accordance with the manufacturer instructions using a Tecan Infinite M1000 plate reader with Tecan i-Control 3.1.9.0 software.

HeLa cells utilized in this study were grown under the same growing conditions and using the same media as described for the NTM5 cells.

### De-glycosylation experiment and Western blot conditions

For Western blot, the doublet appearance of secreted human MYOC has been attributed to *N-*glycosylation [20]. PNGase F (Peptide-*N*-Glycosidase F) is an amidase that cleaves between GlcNAc and asparagine residues of *N*-linked glycoproteins and is the most effective method for removal of *N-*linked oligosaccharides from glycoproteins [58]. We wanted to see if mouse MYOC protein was glycosylated similarly to human MYOC. For this experiment, extracts from NTM5 were utilized. For the de-glycosylation experiment, 20μg cell lysates were treated with PNGase F (NEB, P07004S) according to manufacturer instructions. 5X SDS loading buffer (0.25M Tris, pH 7.0, 40% Glycerol, 8% SDS, 20% beta-mercaptoethanol, 0.1% Bromophenol Blue) was added to samples and Western blot analysis completed using 10% SDS-PAGE gels (Bio-Rad Mini-PROTEAN TGX Gels, 456–1034) in the Bio-Rad Mini-Protean Tetra System followed by wet transfer to PVDF membranes (Millipore, IPVH00010). Membranes were blocked using a solution of 5% non-fat milk in 1X TBS-T. Membranes were treated with primary antibody (1:1000 in a solution of 1% non-fat milk and 1X TBS-T) with gentle rocking over-night at 4°C. Membranes were washed with 1X TBS-T followed by 90 minute room temperature incubation with species appropriate alkaline phosphatase (AP)-conjugated secondary antibody (Abcam). Following 1X TBS-T washes, membranes were exposed to ECF substrate (VWR, RPN5785) and imaged using a Bio-Rad GelDoc XR+ Imaging System with Image Lab 5.2.1 software. Densitometry for quantification of western blots was performing using the GelDoc software.

As a loading control, membranes were stripped according to manufacturer instructions using 1X Strong Stripping Buffer (Millipore, 2504) and after blocking in a 5% non-fat milk 1X TBS-T solution the membranes were treated with anti-GAPDH.

### Isolation of soluble, insoluble, and secreted protein fractions

NTM5 cells in 10cm culture dishes were transiently-transfected as described. The control vector was CMV-Tag1 (Aligent, 211170) and the CMV-MYOC plasmid cDNAs was constructed and sequenced by GeneWiz (Cambridge, MA). One plasmid contained cDNA for normal (wild-type) untagged human MYOC (Accession NM_000261) and the other plasmid had cDNA for MYOC with the Y437H mutation. 24 hours post-transfection, cells were washed five times with 1X PBS and 10mL serum-free DMEM added to each plate. 48 hours post-transfection cell fractions were collected/harvested. Serum-free media was centrifuged at room temperature for 5 minutes at 1000rpm to remove any debris and supernatants were concentrated 100X using cold centrifugation and Ambion Ultra-4 Centrifugal Filters (Millipore, UFC801008). Sample protein concentration was determined by the Bio-Rad DC protein assay and samples were analyzed by Western blot. For Western blot, 5X SDS loading buffer was added to each sample.

To isolate soluble protein fractions, a minimal amount of RIPA buffer with protease inhibitors was added to each plate of cells and the plates were placed on ice for ten minutes. Cells were scraped from the plates using a cell scraper (Corning, 3008) and samples were centrifuged at 4°C for 10 minutes at 7500rpm. The supernatant was collected as the soluble fraction. Bio-Rad DC protein assay of these samples was completed. 5X SDS loading buffer was added to each sample and samples were placed in a boiling water bath for five minutes.

To isolate insoluble cell fractions, 250μL RIPA buffer with protease inhibitors was added to each cell pellet and these samples were briefly sonicated three times (Misonix XL-2000, 7 volts). 100μL of 5X SDS loading buffer was added to each of these samples and the tubes were placed in a boiling water bath for 5 minutes. RIPA buffer was added to the insoluble samples to equalize the volume to that in the soluble fraction samples. Western blot analysis of all NTM5 sample fractions was completed as previously described and the Bio-Rad GelDoc XR+ image software utilized for densitometry of Western blot bands.

### Isolation of *in vivo* protein

Tissue harvested from mice was minced with scissors and homogenized (Omni Tissue Master 125 Homogenizer, Omni International) in RIPA buffer with Roche Complete protease inhibitors. The tissue samples were sonicated using a Misonix Sonicator XL-2000 series with ultrasonic converter (Serial C6498) set at Power setting 1 (5 volts). Samples were cold centrifuged and supernatants saved. Sample protein concentration was determined by the Bio-Rad DC protein assay. For Western blot, 5X SDS loading buffer was added to each sample and samples were placed in a boiling water bath for five minutes before being loaded into wells of 10% SDS-PAGE gels.

### Antibodies

All primary antibodies were from commercial sources. With the exception of anti-GAPDH (1:10000) and anti-DYSF (1:250), all primary antibodies were utilized at a 1:1000 dilution in 1% non-fat milk and 1X TBS-T. All primary antibody incubations were over-night at 4°C with gentle rocking. Primary antibodies were: BiP (Cell Signaling Technology, 3183S), Caspase 3 (Abcam ab13847), Caspase 12 (Abcam, ab18766), CHOP (Abcam, ab11419), CALR (Cell Signaling Technology, 2891S), CKM (Abcam, ab174672), DYSF (Abcam, ab124684), FLAG (Sigma, F1804), GAPDH (Fitzgerald, 10R-G109a; 1:10000), GRP94 (Cell Signaling Technology, 2104S), MYOC (R&D Systems, AF2537; 1μg/μL), MYOC (Origene, TA323708), MYOC (Acris, AP10162PU-N), MYOM1 (Abcam, ab205618), MYOM2 (Abcam, ab93915), MYOM3 (Proteintech, 7692-I-AP), Obscurin (Millipore, ABT160), MURF1/TRIM63 (Abcam, ab172479), SQSTM/p62 (Cell Signaling Technology, 5114S), and Ubiquitin (Abcam, ab134953). All secondary antibodies were AP-conjugated and were utilized at a 1:2000 dilution in 1% non-fat milk and 1X TBS-T. All secondary antibodies were from Abcam (ab97107, ab97237, ab6722).

### Electron microscopy

Mouse gastrocnemius muscle was harvested from 4 to 6 month old mice and immediately placed in fresh 1/2 Karnovsky's Fixative (2% paraformaldehyde + 2.5% glutaraldehyde in 0.08M sodium cacodylate buffer + CaCl_2_; pH 7.4) for two hours at room temperature. The samples were then moved to 4°C for 18 to 24 hours. After this time, each tissue was trimmed so that an approximate 2mm square piece of tissue was obtained. This small piece of tissue was placed in 0.1 M sodium cacodylate buffer (pH 7.4) in flat bottom glass vials and stored at 4°C. Electron microscopy work was completed by an Electron Microscopy Specialist at Schepens Eye Research Institute (SERI) of Massachusetts Eye and Ear Infirmary.

### Cross-sectional area of gastrocnemius muscle

Mouse muscles harvested from 4 to 6 month old female mice were fixed in 10% neutral buffered formalin for 48 hours, dehydrated with graded ethanol, and embedded in paraffin by Tissue-Tek VIP processor (Sakura). Sections of 5μm thickness were stained with Haematoxylin and Eosin (H&E) in Tissue-Tek Prisma (Sakura) and mounted in Tissue-Tek Glas (Sakura). Slides were scanned using an Aperio AT2 scanner (Leica). Images collected and quantitatively analyzed using Halo v2.1 software (Indica Labs). The total number of muscle fibers in 1x10^5^μm^2^ cross-sectional areas of H&E stained gastrocnemius muscle were counted.

### Immunoprecipitation

NTM5 cells in 10cm culture dishes were transiently-transfected as previously described. The control vector was CMV-Tag1 (Aligent, 211170) and the plasmid cDNA was for wild-type untagged MYOC (Accession NM_000261). For immunoprecipitation (IP), tubes were prepared containing 10μg CKM-FLAG protein (Origene, TP302721) along with IP buffer (20mM Tris-HCl pH 7.5, 150mM NaCl, 1mM EDTA, 1mM EGTA, 1% Triton, 2.5mM NaDOC) with Roche Complete protease inhibitors. 50μL of anti-FLAG antibody was added to each tube and tubes were incubated over-night at 4°C with gentle rotation. Later, 40μL Pierce Protein A/G Agarose (ThermoScientific, 20241) beads were added to each tube for 90 minute incubation at 4°C with gentle rotation. Samples were centrifuged (1000xg at 4°C for 3 minutes) to pellet the A/G beads. Beads were washed five times with the IP buffer and then 15μL of 5X SDS Loading buffer added. Sample tubes were placed in a boiling water bath for five minutes and then centrifuged at room temperature at 13000rpm for 3 minutes. The supernatant was retained for Western blot analysis. This IP protocol is a modified version of that provided by Cell Signaling Technology (http://www.cellsignal.com) for native proteins.

## Supporting information

S1 Fig The CMV-MYOC-Y437H transgenic (Tg) mice were found not to have IOP different from the wt mice. *Top—*IOP was monitored for equal numbers of male and female mice that were 4 to 8 months of age for several weeks in the AM and PM hours. IOP data is representative of several experiments for different aged cohorts of animals that were monitored for several weeks. Minimal N = 6 animals per group. *Bottom–*IOP data obtained over several weeks was averaged and summarized. SD is indicated; t-test, p>0.1. Abbreviations–wild-type, wt; transgenic, Tg.
(PDF)

S2 Fig The CMV-MYOC-Y437H aged transgenic (Tg) mice were found not to have axon loss when compared to wt mice. *Top—*Cartoon figure depicting nine 110μm x 82μm rectangle areas in the optic nerve cross section that was sampled for axon quantification. *Middle and Bottom—*Representative images of the optic nerve that were used to determine axon numbers in wt and CMV-MYOC-Y437H transgenic (Tg) animals older than one year of age. Approximately equal numbers of male and females were included in each group, N = 5 wt and N = 12 MYOC Y437H transgenic; t-test, p = 0.98. Abbreviations–wild-type, wt; transgenic, Tg.
(PDF)

S3 Fig Western blots for MYOC and endoplasmic reticulum proteins in CMV-MYOC-Y437H transgenic mouse eye lysates. (**A**) Western blot for human MYOC (using R&D Systems anti-MYOC antibody) in adult mouse whole eye lysates showed no expression of the transgene in the eye. Loading was 40μg tissue lysate per well of a 10% SDS-PAGE gel. In this western blot the total mouse N = 6 and each sample lane represents lysates from different animals. (**B**) Western blots for MYOC and ER proteins using lysates from pooled anterior eye tissue samples [sclera and limbal ring/trabecular meshwork (TM)] isolated from several wt and several CMV-Y437H-MYOC adult transgenic mice. This Western blot for MYOC used a combination of anti-MYOC antibodies [1:500 each of Origene anti-MYOC (TA323708) and Acris anti-MYOC (AP10162PU-N)] which cross-react with mouse and human MYOC. Abbreviations–wild-type, wt; transgenic, Tg; trabecular meshwork, TM.
(PDF)

S4 Fig RT-PCR for mouse Myoc and human MYOC using RNA isolated from wt, CMV-Y437H-MYOC, wt MYOC BAC, and mutant Q368X MYOC BAC gastrocnemius muscle. Tissue samples were from female mice aged 4 to 6 months. Data has been normalized to mouse Gapdh and results indicate a similar transcript level of mouse Myoc for all the mice. The CMV-MYOC-Y437H transgenic had a C_T_ value for human MYOC six cycles earlier than that for mouse Myoc. +/- SD is indicated. Abbreviations–not detected, ND.
(PDF)

S5 Fig Dysf transcript level in wt and CMV-MYOC-Y437H transgenic mice was examined. *Top—*Image is of a 0.8% agarose / 1X TAE gel loaded with PCR product samples. Reverse-transcriptase PCR for Dysf shows amplification of the wt Dysf band (~500bp) while no PCR product is observed for the mutated form of Dysf (~329bp). N = 6 different animals aged 4 to 6 months with a male and female representative for each of the three mouse lines. *Bottom—*Real-time PCR (RT-PCR) results using RNA isolated from wt and CMV-MYOC-Y437H transgenic mice. C_T_ values between the two groups was similar with no statistically significant differences. +/- SD; t-test p = 0.1. Abbreviations–wild-type, wt; transgenic, Tg.
(PDF)

S6 Fig DYSF protein expression in wt and CMV-MYOC-Y437H transgenic mice was examined. Western blot showing DYSF protein expression in gastrocnemius muscle lysates from wt mice of two different backgrounds as well as from the CMV-MYOC-Y437H transgenics. Similar DYSF protein expression was observed for all animals. Arrow indicates predicted size (~238kDa) of mouse DYSF protein. Western blots were stripped and probed with anti-GAPDH to serve as a loading control. N = 6 different animals aged 4 to 6 months with a male and female representative for each of the three mouse lines. Abbreviations–wild-type, wt; transgenic, Tg.
(PDF)

S7 Fig Skeletal muscles and hearts from C57 wt, wt MYOC BAC transgenic, and mutant Q368X MYOC BAC transgenics were harvested and weighted. Weights of gastrocnemius muscle and heart did differ not among the wt and the BAC transgenic groups. The weight of the diaphragm of the mutant Q368X MYOC BAC transgenic was approximately 30% less than the other animals and * represents t-test p<0.001. All tissue samples were from female mice aged 4 to 6 months. N per group is ≥ 4 and +/- SD is indicated. Abbreviations–wild-type, wt; transgenic, Tg.
(PDF)

S8 Fig Electron micrographs of gastrocnemius muscle from 4 to 6 month old female C57 wt, wt MYOC BAC transgenic and mutant Q368X MYOC BAC transgenic mice. Images show that the sarcomeres of C57 and wt MYOC BAC transgenic were very similar with a distinct and prominent M band. In comparison, the M-band in the mutant Q368X MYOC transgenic was faint and appeared dispersed. Direct magnification was 18500X.
(PDF)

S9 Fig Examination of cross-sections of gastronemius muscle from 4 to 6 month old female C57 wt mice, wt MYOC BAC mice, and mutant Q368X MYOC BAC mice did not show differences in muscle fiber size/number. **(A)** Representative images from wt and BAC transgenic mice of 5μm gastrocnemius muscle cross-sections stained with H&E. Each mouse group had ≥ N = 3 mice per group. **(B)** The total number of muscle fibers in 1x10^5^μm^2^ cross-sectional areas of H&E stained gastrocnemius muscle were counted using Halo software. Data is shown +/- SD.
(PDF)
