# Supplementary figures and images for: Mutant myocilin impacts sarcomere ultrastructure in mouse gastrocnemius muscle
Source: PLoS One. 2018 Nov 5;13(11):e0206801. doi: 10.1371/journal.pone.0206801 (PMC6218065; doi:10.1371/journal.pone.0206801)

### Experimental duration

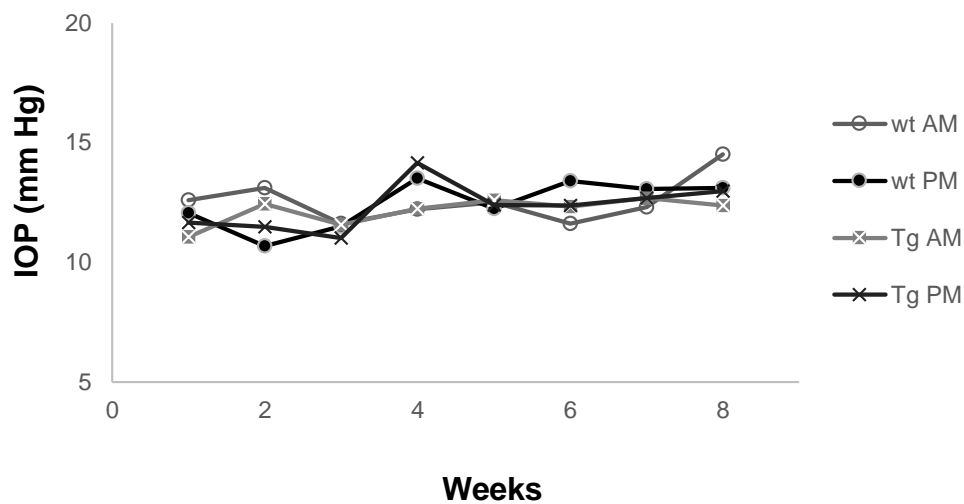

### Summary

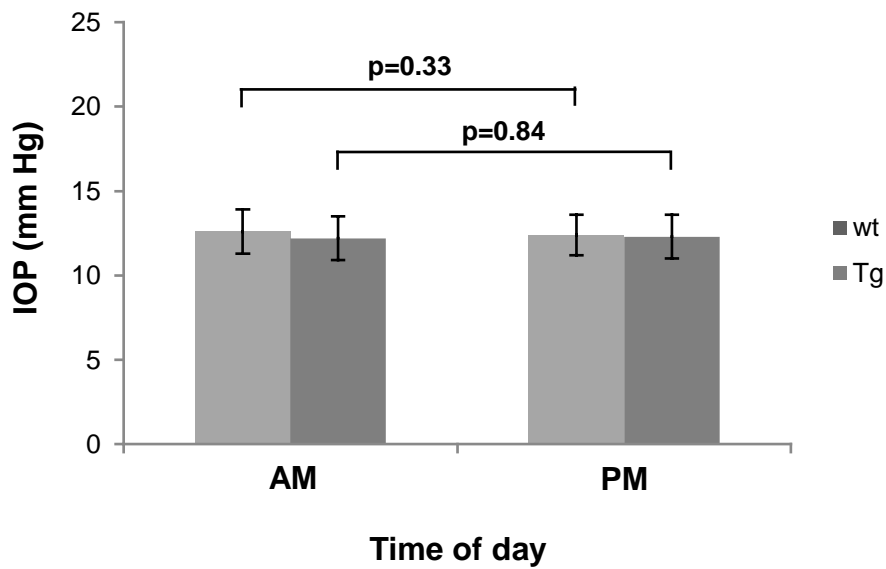

S1 Fig

Supplement: S1 Fig — Top—IOP was monitored for equal numbers of male and female mice that were 4 to 8 months of age for several weeks in the AM and PM hours. IOP data is representative of several experiments for different aged cohorts of animals that were monitored for several weeks. Minimal N = 6 animals per group. Bottom–IOP data obtained over several weeks was averaged and summarized. SD is indicated; t-test, p>0.1. Abbreviations–wild-type, wt; transgenic, Tg. (PDF) [file pone.0206801.s001.pdf]

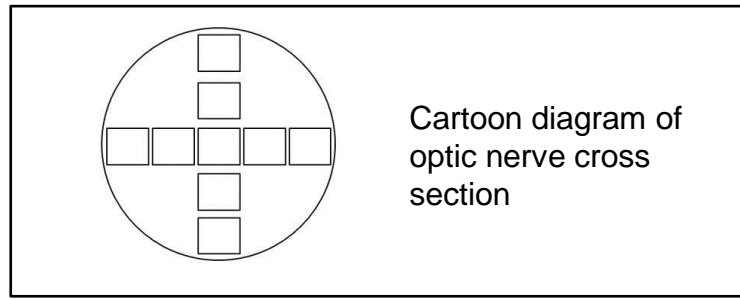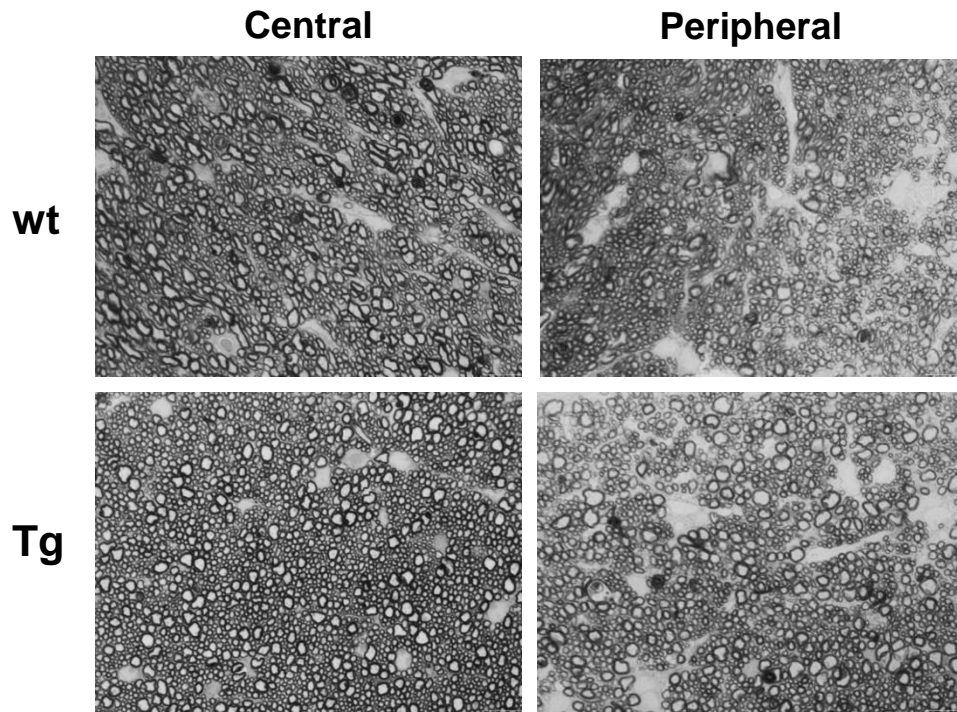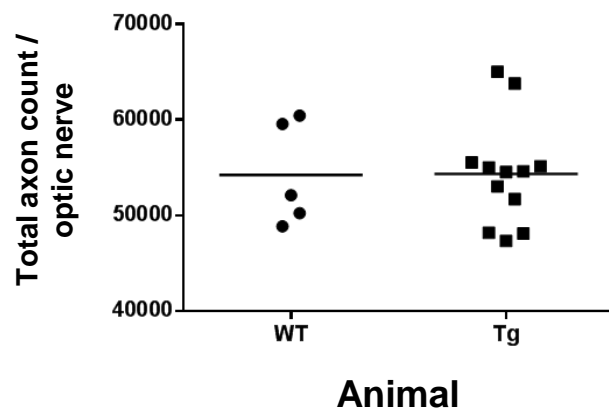

**S2 Fig**

Supplement: S2 Fig — Top—Cartoon figure depicting nine 110μm x 82μm rectangle areas in the optic nerve cross section that was sampled for axon quantification. Middle and Bottom—Representative images of the optic nerve that were used to determine axon numbers in wt and CMV-MYOC-Y437H transgenic (Tg) animals older than one year of age. Approximately equal numbers of male and females were included in each group, N = 5 wt and N = 12 MYOC Y437H transgenic; t-test, p = 0.98. Abbreviations–wild-type, wt; transgenic, Tg. (PDF) [file pone.0206801.s002.pdf]

**A**

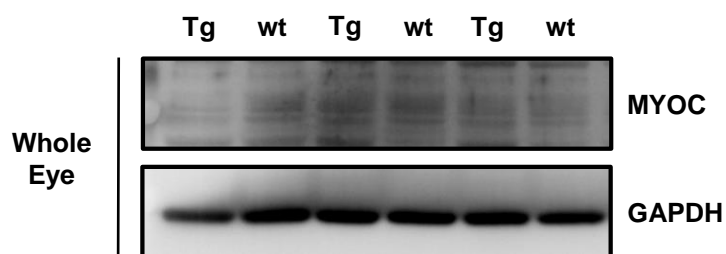

**B**

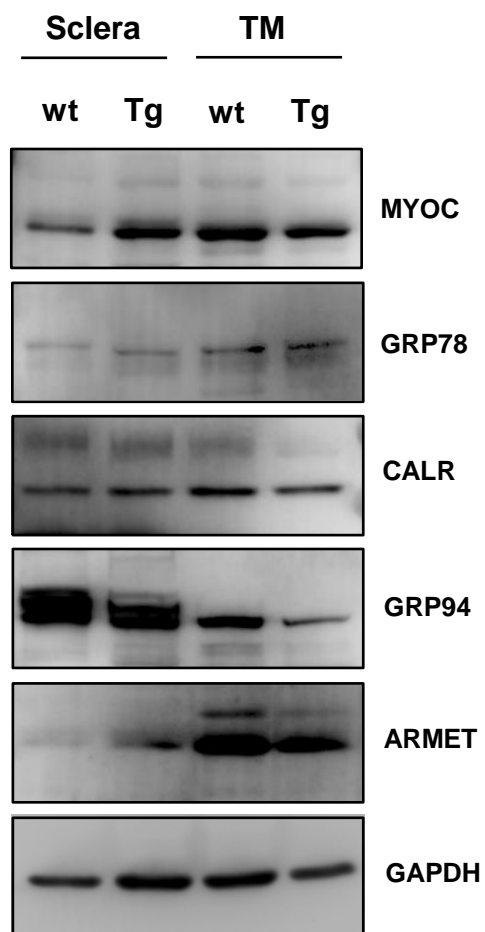

**S3 Fig**

Supplement: S3 Fig — (A) Western blot for human MYOC (using R&D Systems anti-MYOC antibody) in adult mouse whole eye lysates showed no expression of the transgene in the eye. Loading was 40μg tissue lysate per well of a 10% SDS-PAGE gel. In this western blot the total mouse N = 6 and each sample lane represents lysates from different animals. (B) Western blots for MYOC and ER proteins using lysates from pooled anterior eye tissue samples [sclera and limbal ring/trabecular meshwork (TM)] isolated from several wt and several CMV-Y437H-MYOC adult transgenic mice. This Western blot for MYOC used a combination of anti-MYOC antibodies [1:500 each of Origene anti-MYOC (TA323708) and Acris anti-MYOC (AP10162PU-N)] which cross-react with mouse and human MYOC. Abbreviations–wild-type, wt; transgenic, Tg; trabecular meshwork, TM. (PDF) [file pone.0206801.s003.pdf]

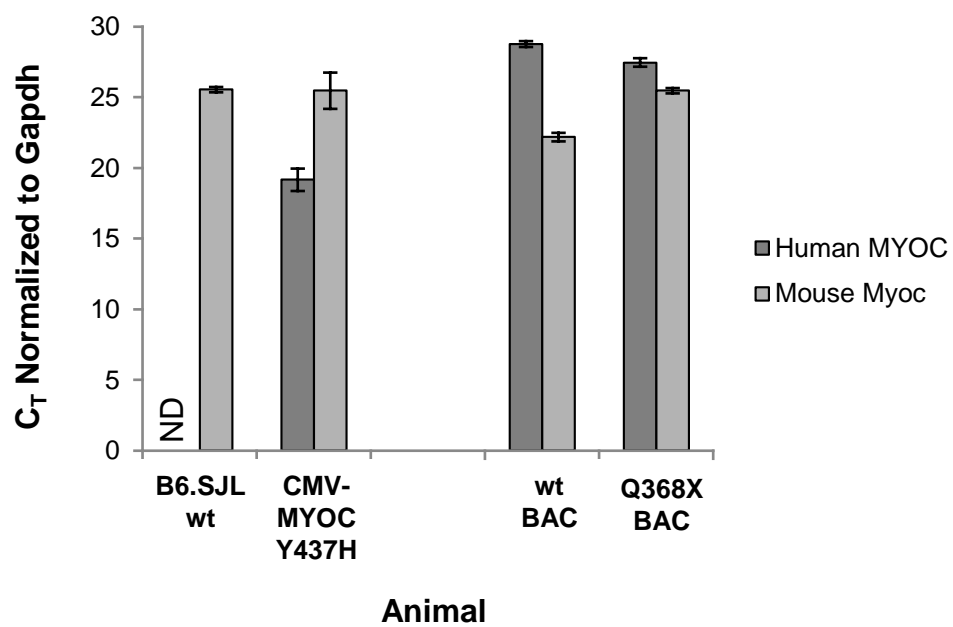

**S4 Fig**

Supplement: S4 Fig — Tissue samples were from female mice aged 4 to 6 months. Data has been normalized to mouse Gapdh and results indicate a similar transcript level of mouse Myoc for all the mice. The CMV-MYOC-Y437H transgenic had a CT value for human MYOC six cycles earlier than that for mouse Myoc. +/- SD is indicated. Abbreviations–not detected, ND. (PDF) [file pone.0206801.s004.pdf]

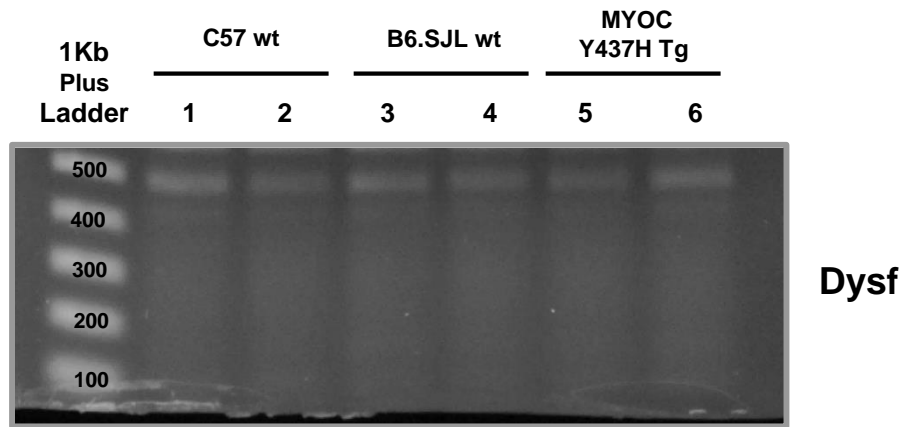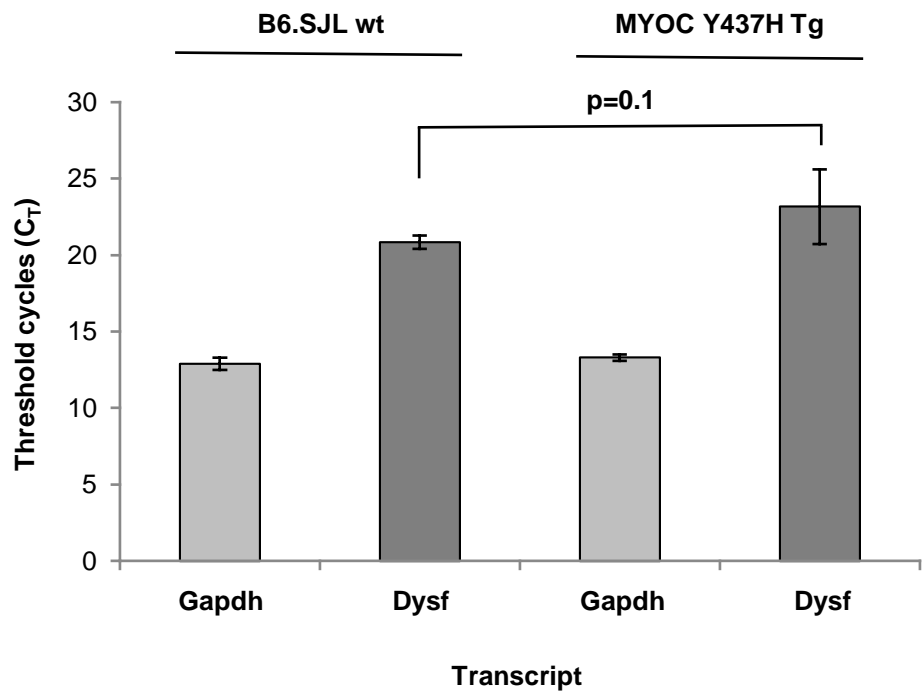

**S5 Fig**

Supplement: S5 Fig — Top—Image is of a 0.8% agarose / 1X TAE gel loaded with PCR product samples. Reverse-transcriptase PCR for Dysf shows amplification of the wt Dysf band (~500bp) while no PCR product is observed for the mutated form of Dysf (~329bp). N = 6 different animals aged 4 to 6 months with a male and female representative for each of the three mouse lines. Bottom—Real-time PCR (RT-PCR) results using RNA isolated from wt and CMV-MYOC-Y437H transgenic mice. CT values between the two groups was similar with no statistically significant differences. +/- SD; t-test p = 0.1. Abbreviations–wild-type, wt; transgenic, Tg. (PDF) [file pone.0206801.s005.pdf]

**F**

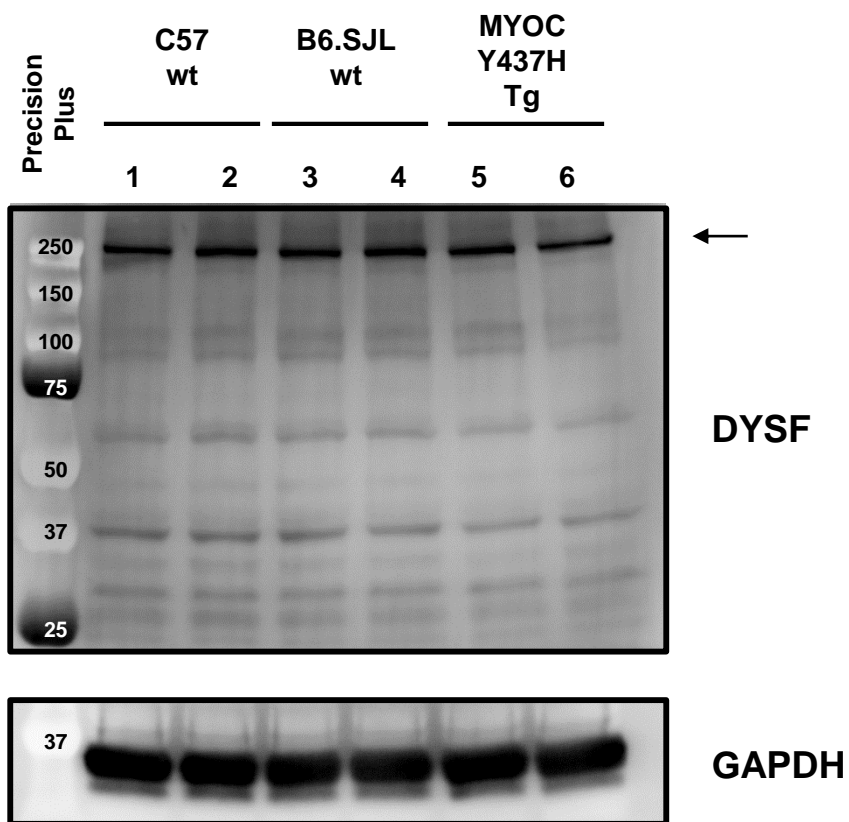

**S6 Fig**

Supplement: S6 Fig — Western blot showing DYSF protein expression in gastrocnemius muscle lysates from wt mice of two different backgrounds as well as from the CMV-MYOC-Y437H transgenics. Similar DYSF protein expression was observed for all animals. Arrow indicates predicted size (~238kDa) of mouse DYSF protein. Western blots were stripped and probed with anti-GAPDH to serve as a loading control. N = 6 different animals aged 4 to 6 months with a male and female representative for each of the three mouse lines. Abbreviations–wild-type, wt; transgenic, Tg. (PDF) [file pone.0206801.s006.pdf]

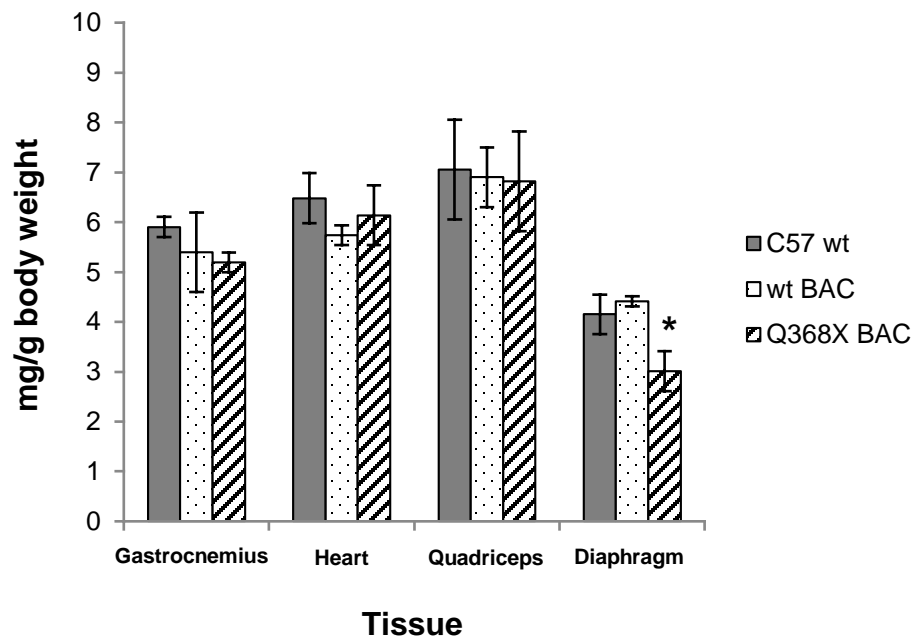

**S7 Fig**

Supplement: S7 Fig — Weights of gastrocnemius muscle and heart did differ not among the wt and the BAC transgenic groups. The weight of the diaphragm of the mutant Q368X MYOC BAC transgenic was approximately 30% less than the other animals and * represents t-test p<0.001. All tissue samples were from female mice aged 4 to 6 months. N per group is ≥ 4 and +/- SD is indicated. Abbreviations–wild-type, wt; transgenic, Tg. (PDF) [file pone.0206801.s007.pdf]

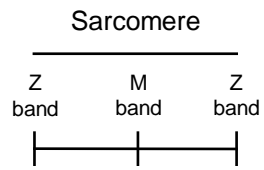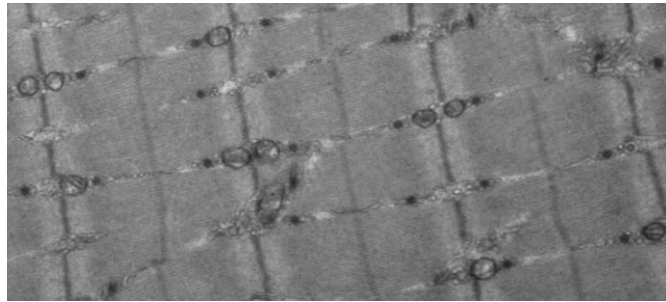

**C57  
wt**

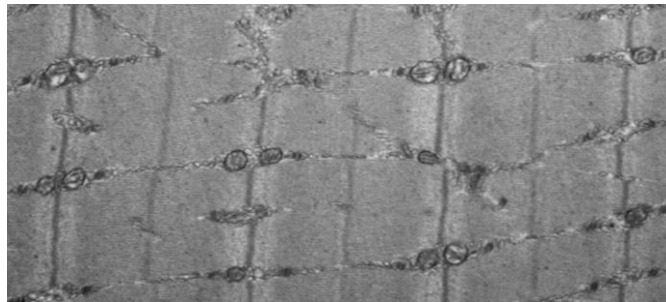

**wt  
MYOC BAC**

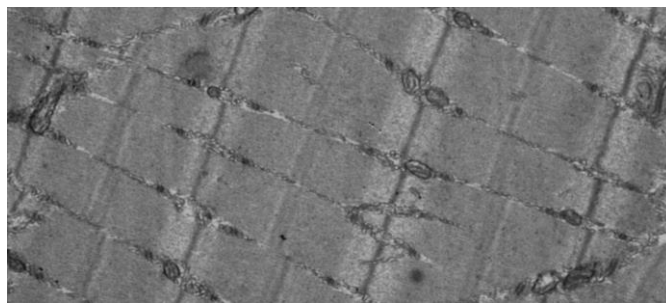

**Q368X  
MYOC BAC**

500 nm

**S8 Fig**

Supplement: S8 Fig — Images show that the sarcomeres of C57 and wt MYOC BAC transgenic were very similar with a distinct and prominent M band. In comparison, the M-band in the mutant Q368X MYOC transgenic was faint and appeared dispersed. Direct magnification was 18500X. (PDF) [file pone.0206801.s008.pdf]

**A**

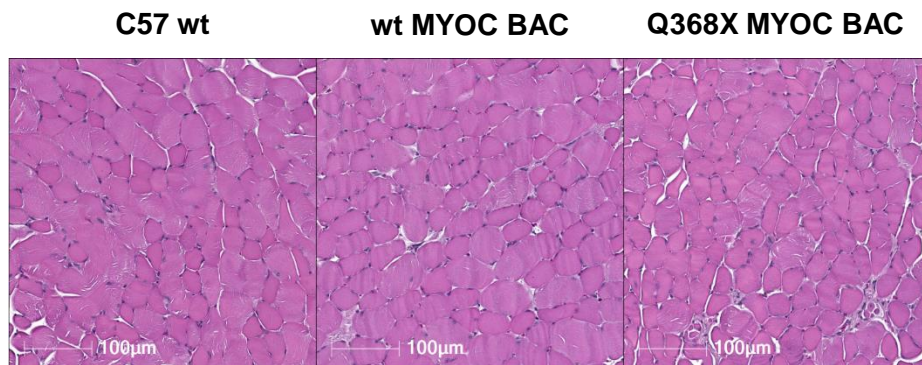

**B**

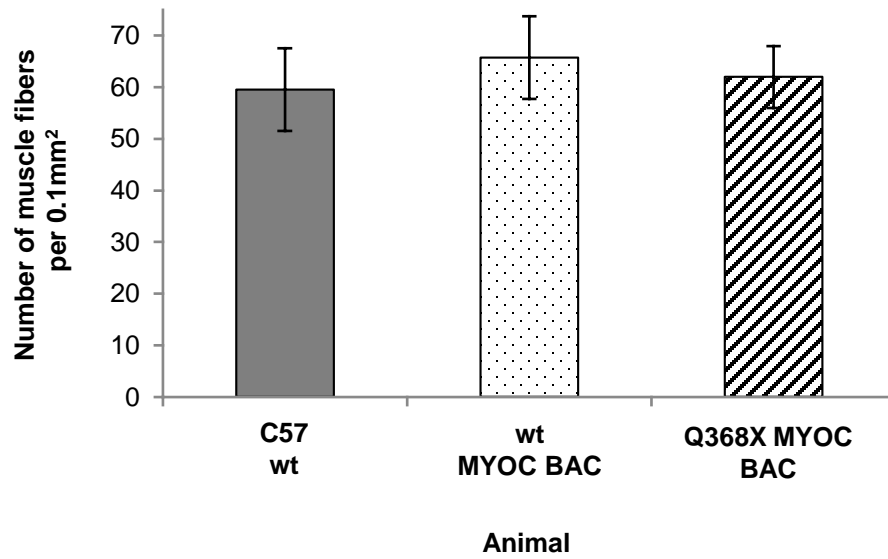

**S9 Fig**

Supplement: S9 Fig — (A) Representative images from wt and BAC transgenic mice of 5μm gastrocnemius muscle cross-sections stained with H&E. Each mouse group had ≥ N = 3 mice per group. (B) The total number of muscle fibers in 1x10^5^μm2 cross-sectional areas of H&E stained gastrocnemius muscle were counted using Halo software. Data is shown +/- SD. (PDF) [file pone.0206801.s009.pdf]
